# A review of Gabonese gorillas and their pathogens: Diversity, transfer and One Health approach to avoid future outbreaks?

**DOI:** 10.3389/fpara.2023.1115316

**Published:** 2023-03-03

**Authors:** Larson Boundenga, Patrice Makouloutou-Nzassi, Barthelemy Ngoubangoye

**Affiliations:** ^1^ Unité de Recherches en Ecologie de la Santé (URES), Centre Interdisciplinaire de Recherches Médicales de Franceville (CIRMF), Franceville, Gabon; ^2^ Department of Anthropology, University of Durham, Durham, United Kingdom; ^3^ Departement de Biologie et Ecologie Animales, Institut de Recherches en Ecologie Tropicale (IRET), Centre National de Recherche Scientifique et Technologique (CENAREST), Libreville, Gabon; ^4^ Centre de Primatologie, Centre Interdisciplinaire de Recherches Médicales de Franceville (CIRMF), Franceville, Gabon

**Keywords:** gorillas, pathogens diversity, transfers, reservoir, One Health

## Abstract

In Africa, great apes, among which gorillas, are the reservoir of several infectious agents, some of which have zoonotic potential. However, scientific reports summarizing data on the pathogens harbored by some primate species still need to be published for the scientific community, conservation, and public health actors. In the case of Gabon, despite its outstanding biodiversity, particularly in great apes, and the history of outbreaks involving wildlife, there is a lack of reports on pathogens found in some ape species living in the vicinity of the human being. Thus, it is becoming urgent for us to synthesize the available data on pathogens (parasites, bacteria, and viruses) identified in gorillas living in different ecosystems of Gabon to assess the risks for the human population. Therefore, this review article presents the diversity of pathogens identified in gorillas in Gabon, their impact on primates’ health, the cases of transfer between gorillas and humans, and the interest in a One Health approach for prevention and a better understanding of the ecology of gorilla’s diseases infection in Gabon.

## Introduction

Besides habitat loss, climate change, non-native species invasion, and overexploitation, pathogens or infectious diseases (ID) are also recognized as determinant factors that sometimes regulate animal population density as drivers of species extension (Chapman et al., 2005; Smith et al., 2009). However, it must be recognized that the role of pathogens alone in this extinction is subject to debate or controversy because the role of ID in population declines was often considered secondary to other factors (Cunningham et al., 2017). However, since 1999 many reports have been published on disease-driven species extinction (Cunningham et al., 2017): such as the decline of tree snail *P. turgida* due to a microsporidian infection in Polynesia (Cunningham and Daszak, 1998), of one-third of Hawaiian honeycreepers and the slime mould induced decline of eelgrass (*Zostera marina*) beds in the USA, leading to the extinction of the eelgrass limpet (*Lottia alveus*) (Thorne and Williams, 1988; Carlton et al., 1991; Juliano, 1998; Daszak and Cunningham, 1999).

Until recently, the main threats to the African ape population were poaching, habitat loss, and human encroachment. However, as in other mammals, ID (i.e., macro and microparasites) have emerged as a threat of the same magnitude. A diverse array of virulent pathogens threatens wild great ape populations, including the Ebola virus (Walsh et al., 2003; Bermejo et al., 2006; Leendertz et al., 2006), Anthrax (Leendertz et al., 2006), simian immunodeficiency virus (SIV) (Keele et al., 2009), and a variety of human respiratory viruses (Köndgen et al., 2008; Kaur et al., 2008). For instance, the Ebola virus caused an 80% decline in the gorilla and chimpanzee populations on the borders of Gabon and the Republic of Congo between 2001 and 2003 (Leroy et al., 2004). However, Gabon, belonging to the Congo Bassin, one of the most important reservoirs of biological biodiversity, is still home to the richest wildlife and plant communities in Africa, with 20% of them being endemic to the country (Maslin, 2008). Moreover, 40% of the world’s gorillas are thought to live in Gabon (Morgan, 2007). In Moukalaba-Doudou National Park, one of the 13 national parks established in 2002, the abundance of lowland gorillas is evaluated at 6.99 gorillas/km^2^ (Takenoshita and Yamagiwa, 2008), and these gorillas are referred to in this review as Gabonese gorillas. For more than a decade, these gorillas have been the subject of intense research activities that have led to habituation and ecotourism projects. In Moulakaba-Doudou and Loango National Parks, two gorilla habituation projects are being conducted, and gorilla tourism is gradually being introduced (Ando et al., 2008; Boesch et al., 2009; Terada et al., 2021). In addition, Gabonese gorillas are present in primatology center and sanctuaries (Ngoubangoye et al., 2019; Boundenga et al., 2021). All these activities promote and increase contact between humans (researchers, local population, tourists) and gorillas with a high potential for pathogen exchange.

Thus, the objective of this review is to summarize the current knowledge regarding the diversity of pathogens known (enzootic and non-enzootic) to have infected Gabonese gorillas and how, through a One Health approach, we can mitigate the threats to the conservation and public health of these gorilla’s pathogens in Gabon settings.

## Diversity of infectious agents identified in Gabonese gorillas

It is estimated that nearly 60% of infectious diseases of animal origin affect humans (Jones et al., 2008). Indeed, African NHPs, particularly lowland gorillas, are known to harbor a wide diversity of pathogens (Liovat et al., 2009), and the cases of transfer are not uncommon (Apetrei et al., 2004; Devaux et al., 2019; Locarnini et al., 2021).

In the case of Gabon, some gorilla populations living in different Gabonese ecosystems were found harboring pathogens, among which are parasites, bacteria, and viruses.

### Parasites

**Figure 1:**
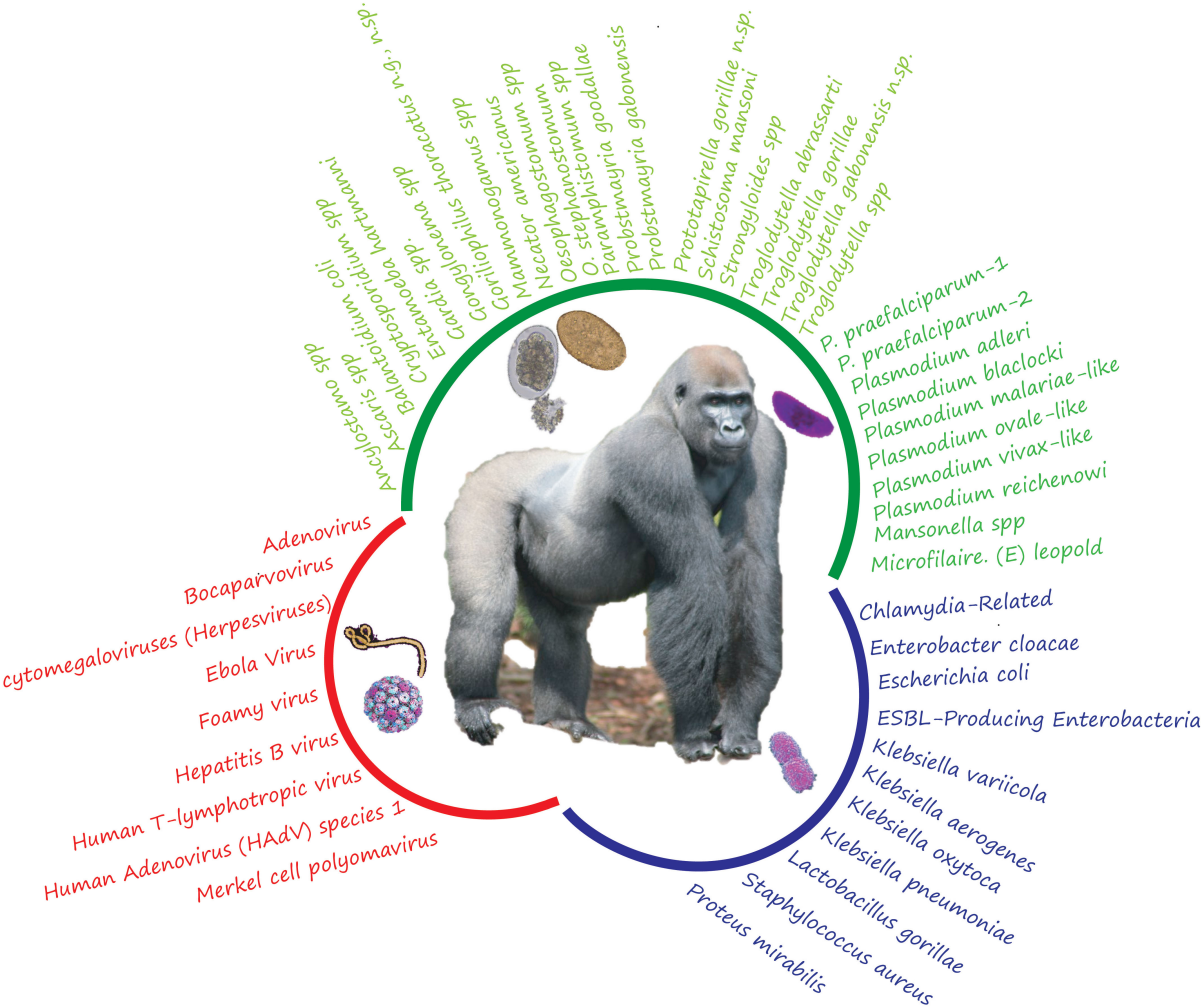
List of different pathogens identified in gabonses gorillas (including parasites, viruses, and bacteria). The different colors indicate the group of pathogens (green for parasites; red: for the virus and blue for the bacteria).

**Figure 2:**
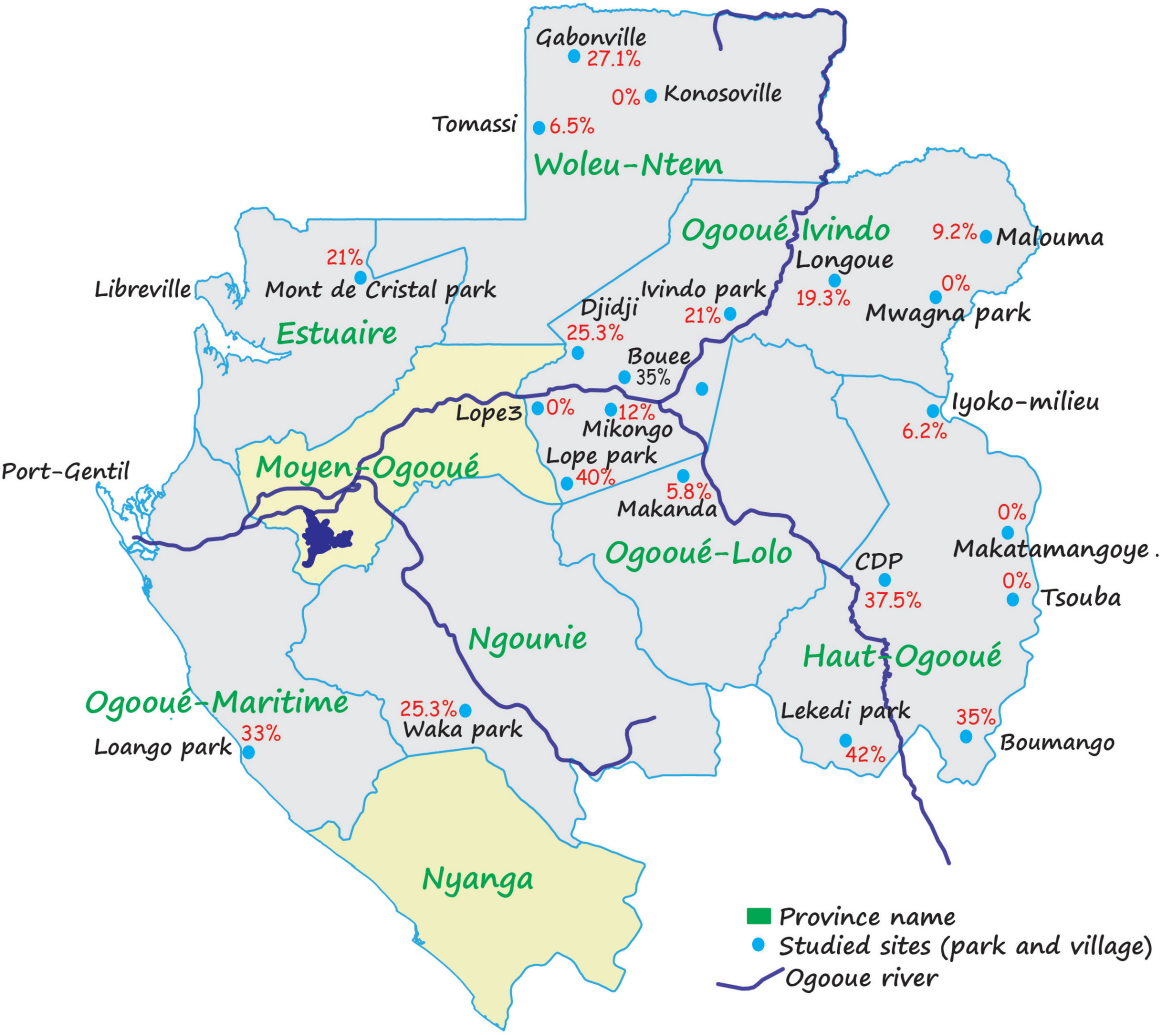
Variation of Plasmodium spp prevalences in Gabon. This picture shows the variations of prevalence within the various populations of gorillas studied (Boundenga et al., 2015).

Concerning parasites, we distinguish between intestinal parasites and malaria parasites. At present, it is estimated that about eight species of malaria parasites have been found in gorillas in the wild or captivity (*Plasmodium praefalciparum 1, Plasmodium praefalciparum 2, Plasmodium gorA* (*Plasmodium adleri*), *Plasmodium gorB* (*Plasmodium blacklocki*), *Plasmodium ovale*-like, *Plasmodium malariae*-like, *Plasmodium vivax*-like, and *Plasmodium reichenowi* which naturally infects chimpanzees) (Prugnolle et al., 2010; Prugnolle et al., 2013; Boundenga et al., 2015; Ngoubangoye et al., 2016) (**Figure 1**). According to the region, the infection rate of gorillas with malaria parasites varies considerably (between 0% and 45%) (for more details see **Figure 2**) (Boundenga et al., 2015). All parasites identified in gorillas belong to two groups: *Laverania* and *Plasmodium*.

The review on intestinal parasites identified almost twenty species of gastrointestinal parasites hosted by the Gabonese gorillas. Thus, from all the studies carried out, regarldless of the technique (corpology or molecular analysis), it was possible to observe (*Ancylostoma* spp.*, Ascaris* spp.*, Balantidium coli, Cryptosporidium* spp.*, Entamoeba hartmanni, Gardia* spp.*, Gongylonema* spp.*, Mammomonogamus* spp.*, Mansonella* spp*, Microfilaria. (E) leopold, Necator americanus, Oesophagostomum stephanostomum, Oesophagostomum* spp.*, Paramphistomum* spp.*, Strongyloides* spp.*, Schistosoma mansoni*, *Troglodytella abrassarti, Troglodytella gorillae, Troglodytella* spp., *Goriliophilus thoracatus* n.g, *Troglodytella gabonensis* n.sp., and *Prototapirella gorillae* n.sp. and several parasites that remain undetermined (Goussard et al., 1983; Imai et al., 1991; Bain et al., 1995; Landsoud-Soukate et al., 1995; Van Zijll Langhout et al., 2010; Hasegawa et al., 2017; Dibakou et al., 2021; Sirima et al., 2021) (**Figure 1**). Several of its species have been identified in gorillas living in different environments.

### Bacteria

8 bacterial genera were identified in Gabonese gorillas and, more precisely, no less than eleven (11) species of bacteria in gorillas living in confined environments (sanctuaries and primate centers) (Nagel et al., 2013; Klöckner et al., 2016) and in wild environments: *Chlamydia-Related*, *Proteus mirabilis* (Klöckner et al., 2016), *Enterobacter cloacae, ESBL-Producing Enterobacteria, Escherichia coli, Klebsiella aerogenes*, *Klebsiella oxytoca*, *Klebsiella pneumoniae, Klebsiella variicola*, (Tsuchida et al., 2018; Mbehang Nguema et al., 2021); *Lactobacillus gorillae* (Tsuchida et al., 2018) and *Staphylococcus aureus* (Nagel et al., 2013) (**Figure 1**).

### Viruses

Finally, several viruses have been recorded in Gabonese gorillas. Indeed, current data show that at least nine virus groups have been detected: Adenovirus (GgorAdV-B7, GgorAdV-B8, GgorAdV-B10, HAdV-B, HAdV-C, or HAdV-E) (Wevers et al., 2010; Hoppe et al., 2015), Human Adenovirus (HAdV) species 1 [(HAdV-B 21(26%), HAdV-C 15(19%), HAdV-F 2 (3%)](Hoppe et al., 2015) (Ebola virus (Leroy et al., 2004; Rouquet et al., 2005; Mombo et al., 2020); Cytomegaloviruses (Herpesviruses: CMV1, CMV2, CMV1 or CMV2) (Murthy et al., 2019); Bocaparvovirus (Nze-Nkogue et al., 2017); Merkel Cell Polyomavirus (Madinda et al., 2016); Human T-lymphotropic virus (Georges-Courbot et al., 1996); Hepatitis B virus (Georges-Courbot et al., 1996); Foamy virus (GorGabColSFV and GorGabOmoSFV) (Campelo et al., 2022) (see **Figure 1**).

**Figure 3:**
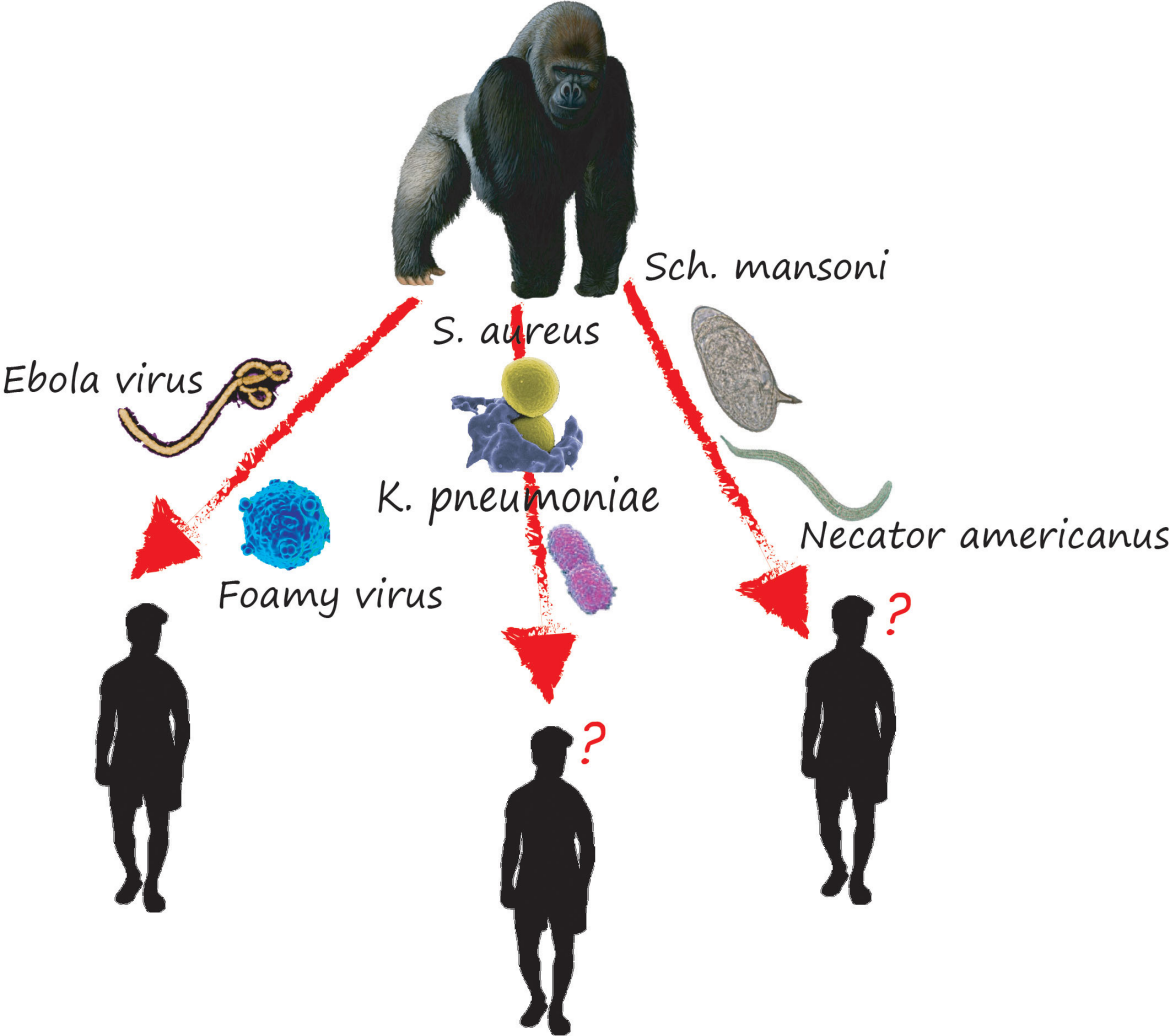
Some examples of the transfer of pathogens between the gorillas and humans and vice versa. In some of the cases illustrated in this picture, gorillas have been clearly identified as the source of the pathogens found in humans in other cases the parasites have been found in both gorillas and humans and the direction of transfer has not yet been identified.

However, the question would be whether the fact that gorillas harbor a wide variety of infectious agents constitutes a risk to human and animal health. We believe that the exchange of viruses might be possible in Gabon settings because some of the viruses isolated are zoonotic, moreover, they were found at variable percentages: 1.9% for Merkel Cell Polyomavirus (Madinda et al., 2016), 30% for HBV (Makuwa et al., 2003), and 48% for HAdV (Hoppe et al., 2015). Therefore, all human activities that favored contact with gorillas infected with one of these viruses would be a potential exposure risk to infectious agents (**Figure 3**). Nevertheless, this remains to be demonstrated by further studies.

## What are the impacts of these infections?

The carriage of these pathogens is not without consequence on the health of the great apes, their population density, and the humans living nearby.

The successive Ebola outbreaks between 2001 and 2003 occurred in the border region of Gabon and the Republic of Congo have decimated approximately 80% of the great ape populations (Huijbregts et al., 2003; Walsh et al., 2003; Leroy and Misson, 2004). For the specific case of Gabon, Leroy et al. (2004) report that they discovered or were informed of 64 animal carcasses (gorillas, chimpanzees, and duikers) over 8 months in the epidemic zone, the Zadié region in Gabon (3000 km^2^) (Leroy et al., 2004). These authors insist that between November and December 2001, at the peak of the epidemic, 36 carcasses of gorillas were found in the area of the epidemic, covering 3000 km^2^. This is likely an underestimate of the severity of the disease, and many more gorillas probably died than were identified. Because the decomposition of a gorilla carcass in the tropical forest lasts about a month, and most of the carcasses were found in the vicinity of villages after 2 hours of walking, hundreds if not thousands of gorillas possibly died from these epidemics (Leroy et al., 2004).

Viruses are not the only ones to cause great apes’ death or deleterious effects on the health of great apes. Indeed, Nagel et al. (2013) reported at the CIRMF primatology center the death of a gorilla having a large necrotizing wound. After analysis, it was septicemia due to *Staphylococcus aureus* (Nagel et al., 2013). Indeed, Nagel et al. reported that molecular analyses revealed that immediate neighboring chimpanzees that were settled to the infected gorillas were infected by the t148 type *S. aureus* known to be virulent (Li et al., 2019). Although mortality of gorillas following *Oesophagostomum* spp. infestation has not yet been reported, the fact remains that recently at the Primatology Center of CIRMF, we observed the death of several chimpanzees following infections with *Oesophagostomum* spp. between 2015 and 2019 (Ngoubangoye et al., 2021). Although not all infections with pathogens may lead to death, they could nevertheless have severe consequences for the health of primates, as was observed during the follow-up of an orphaned youngster in Lékédi Park (Herbert et al., 2015). Thus, all these infections of apes by infectious agents in the wild or captivity are not without consequence and could impact human health if cohabitation with humans favors transfer.

## Are there any transfers, and why?

Cases of potential transmission of pathogens between gorillas and humans, and vice versa, have been reported (Mouinga-Ondémé et al., 2012; Nagel et al., 2013; Prugnolle et al., 2013); (example of transfer of pathogens between gorillas and human **Figure 3**). In the case of simian foamy retroviruses (SFVs), the transmission was done through gorilla bite. Indeed, among the 78 samples from humans screened for SFV, mostly hunters who bitted or scratched by NHPs (gorillas), 19 were SFV seropositive, whose one hunter was infected by gorillas SFV (the PCR confirmed this result) (Mouinga-Ondémé et al., 2012). Regarding *Plasmodium vivax*-like, Prugnolle et al. reported the infection of a tourist who stayed in a forest environment where this parasite is circulated (Prugnolle et al., 2013). Thus, we believe that this tourist would have indeed been infected by the bite of a mosquito with a zoo-anthropophilic feeding behavior (Paupy et al., 2013). All the above demonstrates that Gabonese gorillas are a reservoir for a wide range of infectious agents with zoonotic potential whose transmission is favored by increasing contact (Bittar et al., 2014). However, the question would be whether the existence of such contagious potential would constitute a risk to animal and human health and even, in the long run, hinder the conservation efforts of this species. (Mouinga-Ondémé et al., 2012; Nagel et al., 2013; Prugnolle et al., 2013).

Indeed, infections of human populations with some of these gorillas pathogens have been documented in Gabon. Recently, studies have revealed infection with spumaviruses (Foamy virus) in gorillas and hunters whom gorillas had bitten. The infection of hunters is believed to be the result of frequent contact with these animals’ blood or body fluids (Calattini et al., 2004; Mouinga-Ondémé et al., 2012). The other emblematic example of virus transmission between gorillas and the human population is the infection by the Ebola virus. Indeed, as one of the most virulent infectious agents, the Ebola virus has been responsible for several human epidemics in Gabon due to the direct handling of gorilla and chimpanzee carcasses (Georges-Courbot et al., 1997; Rouquet et al., 2005). During a study of enteroviruses, Mombo et al. (2015) isolated a serotype causing paralysis in great apes (Mombo et al., 2015). Thus, all cases of transmission of infectious agents between great apes, especially gorillas, and humans, result from handling dead animals or permanent cohabitation between these two host groups as described elsewhere (Mekibib and Ariën, 2016).

Concerning gastrointestinal parasites and bacteria, although cases of transfer between gorillas and humans in Gabon have not been demonstrated, several studies report cases of infection of captive and wild gorillas with geohelminths [Sch. Mansoni (Červená et al., 2016), *Necator americanus* (Sirima et al., 2021), *Cryptosporidium* spp (van Zijll Langhout et al., 2010)], and bacteria [*S. aureus* (Nagel et al., 2013), *Chlamydia-Related Bacteria* (Klöckner et al., 2016), *E. coli* (Mbehang Nguema et al., 2021), *K. pneumoniae* (Mbehang Nguema et al., 2021; Shojaei et al., 2022) known to infect humans. However, for some bacteria like *S. aureus*, the origin of transfer has not been identified, i.e. we do not know which the man or the gorillas, transmitted the pathogens to the other.

Furthermore, it is not obvious to believe that the exchange of Plasmodium species between gorillas and humans in the Gabon settings may become frequent insofar as among the vectors responsible for the transmission of simian parasites in gorillas, secondary vectors for the transmission of human malaria in urban and rural areas are found (Paupy et al., 2013; Makanga et al., 2016; Longo-Pendy et al., 2022). For instance*, P. vivax-like*, whose vector species identified are *Anopheles moucheti, Anophles vinckei and Anopheles marshallii* (Prugnolle et al., 2013; Makanga et al., 2016).

This demonstrates the need for more multidisciplinary and longitudinal studies on the real impact of the increase in contact between gorillas and human populations *via* ecotourism activities, habituation, and mining in order to better understand the role of great apes, particularly gorillas, in the transmission or circulation of pathogens in the Gabonese ecosystem.

## How to reconcile conservation and public health in this context of cohabitation?

Wildlife still represents a source of an array of high-impact pathogens that affect human health, with more than 72% of human emerging infectious diseases having wildlife origin (Jones et al., 2008). In the epidemiology of most described zoonoses, wild animals act as primary reservoirs for transmitting zoonotic agents to humans and domestic animals (Taylor et al., 2001). Zoonoses with a wildlife reservoir are typically caused by various bacteria, viruses, and parasites, whereas fungi are unimportant (Biase et al., 2022). Abundant literature documents the spillover of the pathogen of the Great apes to humans and their impact (Hahn et al., 2000; Verdonck et al., 2007; Sharp and Hahn, 2010; Wevers et al., 2010; Betsem et al., 2011; Leroy et al., 2011a; Leroy et al., 2011b). It is well established that malaria parasites, SIV/HIV, or Ebola virus has emerged from great apes to humankind, significantly impacting public health (Georges-Courbot et al., 1996; Keele et al., 2009; Boundenga, 2019).

**Figure 4:**
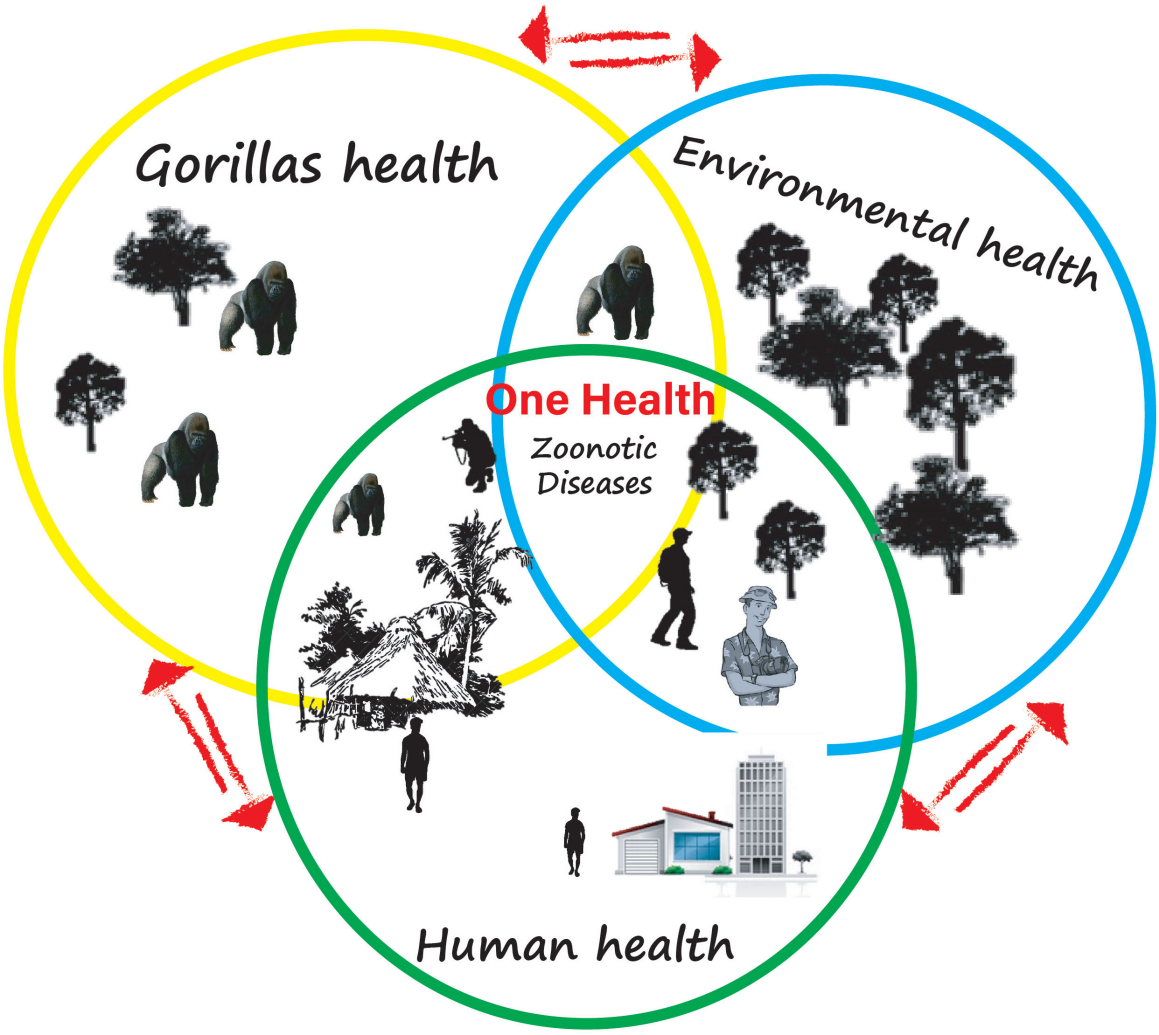
Illustration of the One Health approach. This image illustrates how the increase in human-animal contact, particularly with gorillas in the interface, argues for an increase in the emergence of zoonotic diseases. It shows how in a country such as Gabon where human-gorilla contact is a result of human activities, the success of the best public health prevention strategy would require the collaboration/cooperation of human, animal, and environmental health partners.

These exchanges are the result of human actions on the environment. In this review, we have established that viruses, bacteria, and parasites are capable of zoonotic spread from Gabonese gorillas to humans. It is, therefore, to be feared that with Gabonese government policies aimed at promoting ecotourism and then research and mining activities that will accentuate contacts between wildlife, particularly gorillas, and humans, the exchange of pathogens will be more frequent. This provides ample justification for implementing a One Health approach, mainly since gorilla habituation projects are being conducted in some of Gabon’s thirteen national parks (Hernández Tienda et al., 2022), like in Loango National Park (Oelze et al., 2014; Hernández Tienda et al., 2022) or Moukalaba Doudou National Park (Ando et al., 2008). The One Health approach must prevent and control any emergence, re-emergence, or spread/dissemination of zoonotic pathogens harbored by Gabonese gorillas. To this end, a long-term monitoring system of the health of Gabonese gorillas under habituation must be put in place to achieve what Leendertz et al. (2006) have proposed (Sacks et al., 2018): baseline data on the pathogens of gorillas in Gabon settings. This approach should therefore make it possible to build up a biobank of Gabonese gorilla pathogens, monitor any possible exchange with susceptible animals or humans in direct or indirect contact with these gorillas, and understand the environmental factors have led to this pathogen transfer. In the Gabonese context, with previous Ebola outbreaks that affected human populations, there is an urgent need to implement what Zimmerman et al. (2022) have called “Great Ape Health Watch”, which consist of standardizing surveillance across sites and geographic scales, which monitors primate health in real-time and generates early warnings of disease outbreaks (Zimmerman et al., 2022). In addition, the local population must be educated on the characteristics, ecology, and history of gorilla pathogens and the threats it poses to the wildlife and the human population in case of spillover (Kuisma et al., 2019). They should be aware of how to act when finding gorilla carcasses (even of any animal) to avoid exposure and how to inform local research institutions (CIRMF, IRET/CENAREST) able to conduct investigations to confirm the cause of these deaths. However, for adequate surveillance and effective implementation of a One health approach (**Figure 4**), it is more than necessary to establish multi-sectoral teams that should include all sectors involved in public health surveillance (environmental, research, health, and agricultural services).

## Conclusion

In conclusion, Gabonese gorillas are a reservoir for a wide range of pathogens, some of which are zoonotic with deleterious effects on their health and that of populations living in their vicinity, as cases of exchange have been documented. These pathogens threaten the biodiversity conservation efforts undertaken by the Gabonese authorities in creating national parks (13) to promote ecotourism. There is an urgent need for a real strategy based on a One-health approach to prevent and control any emergence, re-emergence, and transmission of pathogens between Gabonese gorillas and the local population.

## Author contributions

All authors contributed to the article and approved the submitted version.

## References

[B1] Ando C. Iwata Y. Yamagiwa J. (2008). Progress of habituation of western lowland gorillas and their reaction to observers in moukalaba-doudou national park, Gabon. Afr. Study Monographs. Supplementary Issue. 39, 55–69. doi: 10.14989/66238

[B2] Apetrei C. Robertson D. L. Marx P. A. (2004). The history of SIVS and AIDS: Epidemiology, phylogeny and biology of isolates from naturally SIV infected non-human primates (NHP) in Africa. Front. Biosci. 9, 225–254. doi: 10.2741/1154 14766362

[B3] Bain O. Moisson P. Huerre M. Landsoud-Soukate J. Tutin C. (1995). Filariae from a wild gorilla in Gabon with description of a new species of mansonella. Parasite 2, 315–322. doi: 10.1051/parasite/1995023315 8520803

[B4] Bermejo M. Rodríguez-Teijeiro J. D. Illera G. Barroso A. Vilà C. Walsh P. D. (2006). Ebola Outbreak killed 5000 gorillas. Science 314 (5805), 1564–1564. doi: 10.1126/science.1133105 17158318

[B5] Betsem E. Rua R. Tortevoye P. Froment A. Gessain A. (2011). Frequent and recent human acquisition of simian foamy viruses through apes' bites in central Africa. PloS Pathog. 7, e1002306. doi: 10.1371/journal.ppat.1002306 22046126 PMC3203161

[B6] Biase A. G. Albertini T. Z. De Mello R. F. (2022). On supervised learning to model and predict cattle weight in precision livestock breeding. Comput. Electron. Agric. 195, 106706. doi: 10.1016/j.compag.2022.106706

[B7] Bittar F. Keita M. B. Lagier J.-C. Peeters M. Delaporte E. Raoult D. (2014). Gorilla gorilla gorilla gut: A potential reservoir of pathogenic bacteria as revealed using culturomics and molecular tools. Sci. Rep. 4, 1–5. doi: 10.1038/srep07174 PMC4241516 25417711

[B8] Boesch C. Head J. Robbins M. M. (2009). Complex tool sets for honey extraction among chimpanzees in loango national park, Gabon. J. Hum. Evol. 56, 560–569. doi: 10.1016/j.jhevol.2009.04.001 19457542

[B9] Boundenga L. (2019). “Origin of two most virulent agents of human malaria: *Plasmodium falciparum* and *Plasmodium vivax*,” in Malaria. IntechOpen. 1–6. doi: 10.5772/intechopen.84481.

[B10] Boundenga L. Ngoubangoye B. Moukodoum N. Dibakou S. E. Moussadji C. Hugot J. P. (2021). Diversity of parasites in two captive chimpanzee populations in southern Gabon. Infect. Genet. Evol. 91, 104807. doi: 10.1016/j.meegid.2021.104807 33737228

[B11] Boundenga L. Ollomo B. Rougeron V. Mouele L. Y. Mve-Ondo B. Delicat-Loembet L. M.. (2015). Diversity of malaria parasites in great apes in Gabon. Malar J. 14, 111. doi: 10.1186/s12936-015-0622-6 25889049 PMC4364493

[B12] Calattini S. Nerrienet E. Mauclère P. Georges-Courbot M. C. Saïb A. Gessain A. (2004). Natural simian foamy virus infection in wild-caught gorillas, mandrills and drills from Cameroon and Gabon. J. Gen. Virol. 85, 3313–3317. doi: 10.1099/vir.0.80241-0 15483245

[B13] Campelo T. A. Cardoso De Sousa P. R. Nogueira L. L. Frota C. C. Antas P. R. (2022). Corrigendum: Revisiting the methods for detecting mycobacterium tuberculosis: What has the new millennium brought thus far? Access Microbiol. 4, 000294. doi: 10.1099/acmi.0.000294 35252747 PMC8895602

[B14] Carlton J. T. Vermeij G. J. Lindberg D. R. Carlton D. A. Dubley E. (1991). The first historical extinction of a marine invertebrate in an ocean basin: The demise of the eelgrass limpet lottia alveus. Biol. Bull. 180, 72–80. doi: 10.2307/1542430 29303643

[B15] Červená B. Brant S. V. Fairet E. Shirley M. H. Petrželková K. J. Modrý D.. (2016). Schistosoma mansoni in Gabon: Emerging or ignored? Am. J. Trop. Med. Hyg 95, 849–851. doi: 10.4269/ajtmh.16-0446 27503513 PMC5062786

[B16] Chapman C. A. Gillespie T. R. Goldberg T. L. (2005). Primates and the ecology of their infectious diseases: How will anthropogenic change affect host-parasite interactions? Evolutionary Anthropol: Issues News Reviews: Issues News Rev. 14, 134–144. doi: 10.1002/evan.20068

[B17] Cunningham A. A. Daszak P. (1998). Extinction of a species of land snail due to infection with a microsporidian parasite. Conserv. Biol. 12, 1139–1141. doi: 10.1046/j.1523-1739.1998.97485.x

[B18] Cunningham A. A. Daszak P. Wood J. L. (2017). One health, emerging infectious diseases and wildlife: Two decades of progress? Philos. Trans. R. Soc. B: Biol. Sci. 372, 20160167. doi: 10.1046/j.1523-1739.1998.97485.x PMC5468692 28584175

[B19] Daszak P. Cunningham A. (1999). Extinction by infection. Trends Ecol. Evol. 14, 279. doi: 10.1016/S0169-5347(99)01665-1 10370265

[B20] Devaux C. A. Mediannikov O. Medkour H. Raoult D. (2019). Infectious disease risk across the growing human-non human primate interface: A review of the evidence. Front. Public Health 7, 305. doi: 10.3389/fpubh.2019.00305 31828053 PMC6849485

[B21] Dibakou S. E. Maloueki U. Ngoubangoye B. Boundenga L. Ntie S. Tsoumbou T. A.. (2021). Diversity of gastrointestinal parasites in sympatric mammals in moukalaba-doudou national park, Gabon. Vet. World 14, 3149–3155. doi: 10.14202/vetworld.2021.3149-3155 35153406 PMC8829402

[B22] Georges-Courbot M. C. Moisson P. Leroy E. Pingard A. M. Nerrienet E. Dubreuil G.. (1996). Occurrence and frequency of transmission of naturally occurring simian retroviral infections (SIV, STLV, and SRV) at the CIRMF primate center, Gabon. J. Med. Primatol 25, 313–326. doi: 10.1111/j.1600-0684.1996.tb00023.x 9029395

[B23] Georges-Courbot M. C. Sanchez A. Lu C. Y. Baize S. Leroy E. Lansout-Soukate J.. (1997). Isolation and phylogenetic characterization of Ebola viruses causing different outbreaks in Gabon. Emerg. Infect. Dis. 3, 59–62. doi: 10.3201/eid0301.970107 9126445 PMC2627600

[B24] Goussard B. Collet J. Y. Garin Y. Tutin C. E. Fernandez M. (1983). The intestinal entodiniomorph ciliates of wild lowland gorillas (Gorilla gorilla gorilla) in Gabon, West Africa. J. Med. Primatol 12, 239–249. doi: 10.1111/j.1600-0684.1983.tb00080.x 6438333

[B25] Hahn B. H. Shaw G. M. De Cock K. M. Sharp P. M. (2000). AIDS as a zoonosis: Scientific and public health implications. Science 287, 607–614. doi: 10.1126/science.287.5453.607 10649986

[B26] Hasegawa H. Shigyo M. Yanai Y. Mclennan M. R. Fujita S. Makouloutou P.. (2017). Molecular features of hookworm larvae (Necator spp.) raised by coproculture from Ugandan chimpanzees and gabonese gorillas and humans. Parasitol. Int. 66, 12–15. doi: 10.1016/j.parint.2016.11.003 27840196

[B27] Herbert A. Boundenga L. Meyer A. Moukodoum D. N. Okouga A. P. Arnathau C.. (2015). Malaria-like symptoms associated with a natural plasmodium reichenowi infection in a chimpanzee. Malar J. 14, 220. doi: 10.1186/s12936-015-0743-y 26032157 PMC4502519

[B28] Hernández Tienda C. Majolo B. Romero T. Illa Maulany R. Oka Ngakan P. Beltrán Francés V.. (2022). The habituation process in two groups of wild moor macaques (Macaca maura). Int. J. Primatol 43, 291–316. doi: 10.1007/s10764-021-00275-7 35043025 PMC8758468

[B29] Hoppe E. Pauly M. Gillespie T. R. Akoua-Koffi C. Hohmann G. Fruth B.. (2015). Multiple cross-species transmission events of human adenoviruses (HAdV) during hominine evolution. Mol. Biol. Evol. 32, 2072–2084. doi: 10.1093/molbev/msv090 25862141 PMC4833075

[B30] Huijbregts B. De Wachter P. Obiang L. S. N. Akou M. E. (2003). Ebola And the decline of gorilla gorilla gorilla and chimpanzee pan troglodytes populations in minkebe forest, north-eastern Gabon. Oryx 37, 437–443. doi: 10.1017/S0030605303000802

[B31] Imai S. Ikeda S. Collet J. Y. Bonhomme A. (1991). Entodiniomorphid ciliates from the wild lowland gorilla with the description of a new genus and three new species. Eur. J. Protistol 26, 270–278. doi: 10.1016/S0932-4739(11)80148-3 23196284

[B32] Jones K. E. Patel N. G. Levy M. A. Storeygard A. Balk D. Gittleman J. L.. (2008). Global trends in emerging infectious diseases. Nature 451, 990–993. doi: 10.1038/nature06536 18288193 PMC5960580

[B33] Juliano S. A. (1998). Species introduction and replacement among mosquitoes: Interspecific resource competition or apparent competition? Ecology 79, 255–268. doi: 10.1890/0012-9658(1998)079[0255:SIARAM]2.0.CO;2

[B34] Kaur T. Singh J. Tong S. Humphrey C. Clevenger D. Tan W.. (2008). Descriptive epidemiology of fatal respiratory outbreaks and detection of a human-related metapneumovirus in wild chimpanzees (Pan troglodytes) at mahale mountains national park, Western Tanzania. Am. J. Primatol 70, 755–765. doi: 10.1002/ajp.20565 18548512 PMC7159556

[B35] Keele B. F. Jones J. H. Terio K. A. Estes J. D. Rudicell R. S. Wilson M. L.. (2009). Increased mortality and AIDS-like immunopathology in wild chimpanzees infected with SIVcpz. Nature 460, 515–519. doi: 10.1038/nature08200 19626114 PMC2872475

[B36] Klöckner A. Nagel M. Greub G. Aeby S. Hoffmann K. Liégeois F.. (2016). Chlamydia-related bacteria in free-living and captive great apes, Gabon. Emerg. Infect. Dis. 22, 2199–2201. doi: 10.3201/eid2212.150893 27869611 PMC5189123

[B37] Köndgen S. Kühl H. N'goran P. K. Walsh P. D. Schenk S. Ernst N.. (2008). Pandemic human viruses cause decline of endangered great apes. Curr. Biol. 18, 260–264. doi: 10.1016/j.cub.2008.01.012 18222690

[B38] Kuisma E. Olson S. H. Cameron K. N. Reed P. E. Karesh W. B. Ondzie A. I.. (2019). Long-term wildlife mortality surveillance in northern Congo: A model for the detection of Ebola virus disease epizootics. Philos. Trans. R Soc. Lond B Biol. Sci. 374, 20180339. doi: 10.1098/rstb.2018.0339 31401969 PMC6711308

[B39] Landsoud-Soukate J. Tutin C. E. Fernandez M. (1995). Intestinal parasites of sympatric gorillas and chimpanzees in the lopé reserve, Gabon. Ann. Trop. Med. Parasitol. 89, 73–79. doi: 10.1080/00034983.1995.11812931 7741597

[B40] Leendertz F. H. Lankester F. Guislain P. Néel C. Drori O. Dupain J.. (2006). Anthrax in Western and central African great apes. Am. J. Primatol 68, 928–933. doi: 10.1002/ajp.20298 16900500

[B41] Leroy E. Baize S. Gonzalez J. P. (2011a). [Ebola and marburg hemorrhagic fever viruses: Update on filoviruses]. Med. Trop. (Mars) 71, 111–121. 21695865

[B42] Leroy E. M. Gonzalez J. P. Baize S. (2011b). Ebola And marburg haemorrhagic fever viruses: Major scientific advances, but a relatively minor public health threat for Africa. Clin. Microbiol. Infect. 17, 964–976. doi: 10.1111/j.1469-0691.2011.03535.x 21722250

[B43] Leroy P. Misson J. P. (2004). [Epileptic and non-epileptic paroxysmal phenomenons in the child]. Rev. Med. Liege 59, 243–245. 15182037

[B44] Leroy E. M. Rouquet P. Formenty P. Souquière S. Kilbourne A. Froment J. M.. (2004). Multiple Ebola virus transmission events and rapid decline of central African wildlife. Science 303, 387–390. doi: 10.1126/science.1092528 14726594

[B45] Li X. Huang T. Xu K. Li C. Li Y. (2019). Molecular characteristics and virulence gene profiles of staphylococcus aureus isolates in hainan, China. BMC Infect. Dis. 19, 873. doi: 10.1186/s12879-019-4547-5 31640587 PMC6805582

[B46] Liovat A. S. Jacquelin B. Ploquin M. J. Barré-Sinoussi F. Müller-Trutwin M. C. (2009). African Non human primates infected by SIV - why don't they get sick? lessons from studies on the early phase of non-pathogenic SIV infection. Curr. HIV Res. 7, 39–50. doi: 10.2174/157016209787048546 19149553

[B47] Locarnini S. A. Littlejohn M. Yuen L. K. W. (2021). Origins and evolution of the primate hepatitis b virus. Front. Microbiol. 12, 653684. doi: 10.3389/fmicb.2021.653684 34108947 PMC8180572

[B48] Longo-Pendy N. M. Boundenga L. Kutomy P. O. O. Mbou-Boutambe C. Makanga B. Moukodoum N.. (2022). Systematic review on diversity and distribution of anopheles species in Gabon: A fresh look at the potential malaria vectors and perspectives. Pathogens 11 (6), 668–681. doi: 10.3390/pathogens11060668 35745522 PMC9229970

[B49] Madinda N. F. Ehlers B. Wertheim J. O. Akoua-Koffi C. Bergl R. A. Boesch C.. (2016). Assessing host-virus codivergence for close relatives of merkel cell polyomavirus infecting African great apes. J. Virol. 90, 8531–8541. doi: 10.1128/JVI.00247-16 27440885 PMC5021428

[B50] Makanga B. Yangari P. Rahola N. Rougeron V. Elguero E. Boundenga L.. (2016). Ape malaria transmission and potential for ape-to-human transfers in Africa. Proc. Natl. Acad. Sci. U.S.A. 113, 5329–5334. doi: 10.1073/pnas.1603008113 27071123 PMC4868493

[B51] Makuwa M. Onanga R. Souquiere S. Niangui M. Bedjabaga I. Simon F.. (2003). Communications affichees-Resume-Prevalence et diversite des VIH/HTLV et HBV/HCV dans une zone rurale du gabon. le paradoxe de l'Afrique centrale. Bull. la Societe Pathologie Exotique 96, 257–257.

[B52] Maslin J. (2008). Review/Film; saving africa's gorillas. Afr. Stud. Rev. 51, 1–2. Available at: http://www.elegantbrain.com/edu4/classes/readings/254readings/reviews/rev9_gorril.pdf.

[B53] Mbehang Nguema P. P. Onanga R. Ndong Atome G. R. Tewa J. J. Mabika Mabika A. Muandze Nzambe J. U.. (2021). High level of intrinsic phenotypic antimicrobial resistance in enterobacteria from terrestrial wildlife in gabonese national parks. PloS One 16, e0257994. doi: 10.1371/journal.pone.0257994 34637441 PMC8509864

[B54] Mekibib B. Ariën K. K. (2016). Aerosol transmission of filoviruses. Viruses 8.5 (2016), 148–163. doi: 10.3390/v8050148 27223296 PMC4885103

[B55] Mombo I. M. Berthet N. Lukashev A. N. Bleicker T. Brünink S. Léger L.. (2015). First detection of an enterovirus C99 in a captive chimpanzee with acute flaccid paralysis, from the tchimpounga chimpanzee rehabilitation center, republic of Congo. PloS One 10, e0136700. doi: 10.1371/journal.pone.0136700 26301510 PMC4547728

[B56] Mombo I. M. Fritz M. Becquart P. Liegeois F. Elguero E. Boundenga L.. (2020). Detection of Ebola virus antibodies in fecal samples of great apes in Gabon. Viruses 12 (12), 1347–1356. doi: 10.3390/v12121347 33255243 PMC7761173

[B57] Morgan D. (2007). Lignes directrices pour de meilleures pratiques en matière de réduction de l'impact de l'exploitation forestière commerciale sur les grands singes en Afrique centrale. Groupe de spécialistes des primates de la CSE/UICN, Gland, Suisse. IUCN, 34, 1–44. Available at: https://www.primate-sg.org/best_practice_logging/.

[B58] Mouinga-Ondémé A. Caron M. Nkoghé D. Telfer P. Marx P. Saïb A.. (2012). Cross-species transmission of simian foamy virus to humans in rural Gabon, central Africa. J. Virol. 86, 1255–1260. doi: 10.1128/JVI.06016-11 22072747 PMC3255803

[B59] Murthy S. O'brien K. Agbor A. Angedakin S. Arandjelovic M. Ayimisin E. A.. (2019). Cytomegalovirus distribution and evolution in hominines. Virus Evol. 5, vez015. doi: 10.1093/ve/vez015 31384482 PMC6671425

[B60] Nagel M. Dischinger J. Türck M. Verrier D. Oedenkoven M. Ngoubangoye B.. (2013). Human-associated staphylococcus aureus strains within great ape populations in central Africa (Gabon). Clin. Microbiol. Infect. 19, 1072–1077. doi: 10.1111/1469-0691.12119 23398468

[B61] Ngoubangoye B. Boundenga L. Arnathau C. Mombo I. M. Durand P. Tsoumbou T. A.. (2016). The host specificity of ape malaria parasites can be broken in confined environments. Int. J. Parasitol. 46, 737–744. doi: 10.1016/j.ijpara.2016.06.004 27486075

[B62] Ngoubangoye B. Boundenga L. Dibakou S.-E. Tsoumbou T.-A. Moussadji Kinga C. Prugnolle F.. (2021). Surgical treatment of oesophagostomum spp. nodular infection in a chimpanzee at the CIRMF primatology center, Gabon. Case Rep. Veterinary Med. 2021, 1–5. doi: 10.1155/2021/6617416. PMC8019386 33854806

[B63] Ngoubangoye B. Maganga G. D. Boundenga L. Tsoumbou T.-A. Rougeron V. Mombo I. M.. (2019). Absence of paramyxovirus RNA in non-human primate sanctuaries and a primatology center in Gabon. J. Epidemiological Res. 5, 6. doi: 10.5430/jer.v5n2p6

[B64] Nze-Nkogue C. Horie M. Fujita S. Inoue E. Akomo-Okoue E. F. Ozawa M.. (2017). Identification and molecular characterization of novel primate bocaparvoviruses from wild western lowland gorillas of moukalaba-doudou national park, Gabon. Infect. Genet. Evol. 53, 30–37. doi: 10.1016/j.meegid.2017.05.004 28495649

[B65] Oelze V. M. Head J. S. Robbins M. M. Richards M. Boesch C. (2014). Niche differentiation and dietary seasonality among sympatric gorillas and chimpanzees in loango national park (Gabon) revealed by stable isotope analysis. J. Hum. Evol. 66, 95–106. doi: 10.1016/j.jhevol.2013.10.003 24373257

[B66] Paupy C. Makanga B. Ollomo B. Rahola N. Durand P. Magnus J.. (2013). Anopheles moucheti and anopheles vinckei are candidate vectors of ape plasmodium parasites, including plasmodium praefalciparum in Gabon. PloS One 8, e57294. doi: 10.1371/journal.pone.0057294 23437363 PMC3577705

[B67] Prugnolle F. Durand P. Neel C. Ollomo B. Ayala F. J. Arnathau C.. (2010). African Great apes are natural hosts of multiple related malaria species, including plasmodium falciparum. Proc. Natl. Acad. Sci. U.S.A. 107, 1458–1463. doi: 10.1073/pnas.0914440107 20133889 PMC2824423

[B68] Prugnolle F. Rougeron V. Becquart P. Berry A. Makanga B. Rahola N.. (2013). Diversity, host switching and evolution of plasmodium vivax infecting African great apes. Proc. Natl. Acad. Sci. U.S.A. 110, 8123–8128. doi: 10.1073/pnas.1306004110 23637341 PMC3657773

[B69] Rouquet P. Froment J. M. Bermejo M. Kilbourn A. Karesh W. Reed P.. (2005). Wild animal mortality monitoring and human Ebola outbreaks, Gabon and republic of Congo 2001-2003. Emerg. Infect. Dis. 11, 283–290. doi: 10.3201/eid1102.040533 15752448 PMC3320460

[B70] Sacks D. Baxter B. Campbell B. C. V. Carpenter J. S. Cognard C. Dippel D.. (2018). Multisociety consensus quality improvement revised consensus statement for endovascular therapy of acute ischemic stroke. Int. J. Stroke 13, 612–632. doi: 10.1016/j.jvir.2017.11.026 29786478

[B71] Sharp P. M. Hahn B. H. (2010). The evolution of HIV-1 and the origin of AIDS. Philos. Trans. R Soc. Lond B Biol. Sci. 365, 2487–2494. doi: 10.1098/rstb.2010.0031 20643738 PMC2935100

[B72] Shojaei P. Pourmohammadi K. Hatam N. Bastani P. Hayati R. (2022). Identification and prioritization of critical factors affecting the performance of Iranian public hospitals using the best-worst method: A prospective study. Iran J. Med. Sci. 47, 549–557. doi: 10.30476/ijms.2021.91256.2237 36380978 PMC9652491

[B73] Sirima C. Bizet C. Hamou H. Červená B. Lemarcis T. Esteban A.. (2021). Soil-transmitted helminth infections in free-ranging non-human primates from Cameroon and Gabon. Parasit Vectors 14, 354. doi: 10.1186/s13071-021-04855-7 34225777 PMC8259424

[B74] Smith K. F. Acevedo-Whitehouse K. Pedersen A. B. (2009). The role of infectious diseases in biological conservation. Anim. Conserv. 12, 1–12. doi: 10.1111/j.1469-1795.2008.00228.x

[B75] Takenoshita Y. Yamagiwa J. (2008). Estimating gorilla abundance by dung count in the northern part of moukalaba-doudou national park, Gabon. Afr. Study Monographs. Supplementary Issue. 39, 41–54. Available at: https://jambo.africa.kyoto-u.ac.jp/kiroku/asm_suppl/abstracts/pdf/ASM_s39/4Takenoshita.pdf.

[B76] Taylor L. H. Latham S. M. Woolhouse M. E. (2001). Risk factors for human disease emergence. Philos. Trans. R Soc. Lond B Biol. Sci. 356, 983–989. doi: 10.1098/rstb.2001.0888 11516376 PMC1088493

[B77] Terada S. Yobo C. M. Moussavou G.-M. Matsuura N. (2021). Human-elephant conflict around moukalaba-doudou national park in gabon: Socioeconomic changes and effects of conservation projects on local tolerance. Trop. Conserv. Sci. 14, 19400829211026775. doi: 10.1177/19400829211026775

[B78] Thorne E. T. Williams E. S. (1988). Disease and endangered species: The black-footed ferret as a recent example. Conserv. Biol. 2, 66–74. doi: 10.1111/j.1523-1739.1988.tb00336.x

[B79] Tsuchida S. Kakooza S. Mbehang Nguema P. P. Wampande E. M. Ushida K. (2018). Characteristics of gorilla-specific lactobacillus isolated from captive and wild gorillas. Microorganisms 6, 86–96. doi: 10.3390/microorganisms6030086 30110987 PMC6165273

[B80] Van Zijll Langhout M. Reed P. Fox M. (2010). Validation of multiple diagnostic techniques to detect cryptosporidium sp. and giardia sp. in free-ranging western lowland gorillas (Gorilla gorilla gorilla) and observations on the prevalence of these protozoan infections in two populations in Gabon. J. Zoo Wildl Med. 41, 210–217. doi: 10.1638/2009-0051R1.1 20597211

[B81] Verdonck K. González E. Van Dooren S. Vandamme A. M. Vanham G. Gotuzzo E. (2007). Human T-lymphotropic virus 1: recent knowledge about an ancient infection. Lancet Infect. Dis. 7, 266–281. doi: 10.1016/S1473-3099(07)70081-6 17376384

[B82] Walsh P. D. Abernethy K. A. Bermejo M. Beyers R. De Wachter P. Akou M. E.. (2003). Catastrophic ape decline in western equatorial Africa. Nature 422, 611–614. doi: 10.1038/nature01566 12679788

[B83] Wevers D. Leendertz F. H. Scuda N. Boesch C. Robbins M. M. Head J.. (2010). A novel adenovirus of Western lowland gorillas (Gorilla gorilla gorilla). Virol. J. 7, 1–8. doi: 10.1186/1743-422X-7-303 21054831 PMC2989969

[B84] Zimmerman D. M. Mitchell S. L. Wolf T. M. Deere J. R. Noheri J. B. Takahashi E.. (2022). Great ape health watch: Enhancing surveillance for emerging infectious diseases in great apes. Am. J. primatol 84, e23379. doi: 10.1002/ajp.23379 35389523

